# Innovations from the “ivory tower”: Wilhelm Barthlott and the paradigm shift in surface science

**DOI:** 10.3762/bjnano.8.41

**Published:** 2017-02-08

**Authors:** Christoph Neinhuis

**Affiliations:** 1 Institute for Botany, Technische Universität Dresden, 01062 Dresden, Germany

**Keywords:** Wilhelm Barthlott, 70th birthday, self-cleaning surfaces, lotus-effect

## Abstract

This article is mainly about borders that have tremendous influence on our daily life, although many of them exist and act mostly unrecognized. In this article the first objective will be to address more generally the relation between university and society or industry, borders within universities, borders in thinking and the huge amount of misunderstandings and losses resulting from these obvious or hidden borders. In the second part and in more detail, the article will highlight the impact of the research conducted by Wilhelm Barthlott throughout his scientific career during which not only one border was removed, shifted or became more penetrable. Among the various fields of interest not mentioned here (e.g., systematics of Cactaceae, diversity and evolution of epiphytes, the unique natural history of isolated rocky outcrops called inselbergs, or the global distribution of biodiversity), plant surfaces and especially the tremendous diversity of minute structures on leaves, fruits, seeds and other parts of plants represent a common thread through 40 years of scientific career of Wilhelm Barthlott. Based on research that was regarded already old-fashioned in the 1970s and 1980s, systematic botany, results and knowledge were accumulated that, some 20 years later, initiated a fundamental turnover in how surfaces were recognized not only in biology, but even more evident in materials science.

## Separation

**Figure 1:**
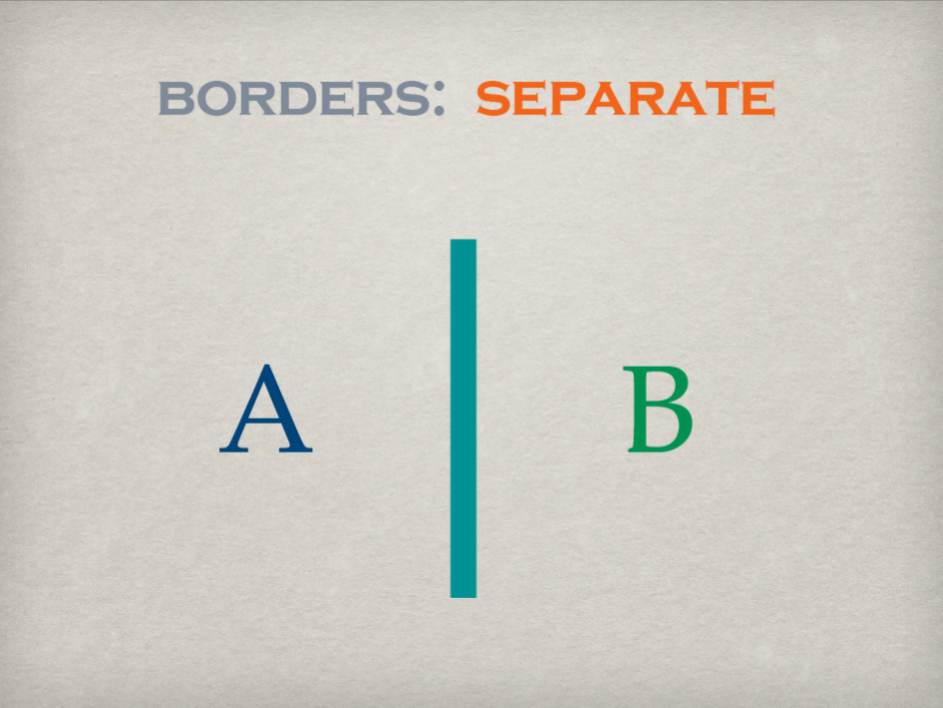
The bar separates A from B, one of the main functions of borders.

Most obviously, borders are meant to separate two or more entities from another (Figure 1). It might be our atmosphere separating us from space, an ocean separating two continents, a door in a building separating two rooms, down to a layer of atoms between a bulk material and its environment. Borders are inevitably necessary as can be seen from the compartmentation of a cell by membranes, essential for the function of all living organisms from archaea to the majestic blue whale, and eventually the biosphere.

On the other hand, borders separate in a sense that two entities are not able to get in contact with each other. Be it for the exchange of matter, energy or information, or to look at or touch something or someone, or to mix or merge two components for the good or the bad.

**Figure 2:**
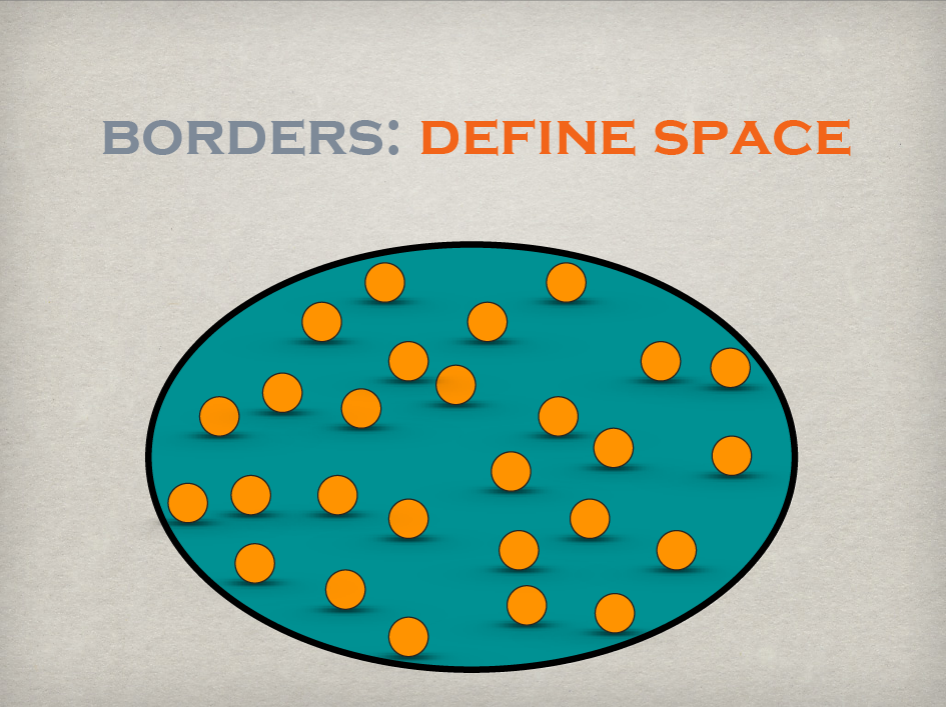
The circle encloses a defined space separated from the surrounding.

In addition, borders define space in which a given activity may take place or not, without affecting the neighbouring space (Figure 2). It may be a playground for children, a research lab, a space station, or a submarine. And in many cases, borders provide shelter or act as a protective cover, enabling individual development, or experimental approaches off the main stream that may or may not be successful, at least not at a given time.

These entities, separated from a dominating larger environment, may serve as incubators fostering new approaches that on the one hand may never be applied or that on the other hand anticipate developments becoming relevant at a much later stage, often disconnected from the original work. We know a large number of inventions, drafts of machinery or theories that were commonly accepted, received attention, or became important only years or even centuries after their conception. In this regard, Leonardo da Vinci is one of the most frequently mentioned names, because he was obviously ahead of his time in many fields of natural history, medicine, and various aspects of engineering. Quite a number of such developments took place in a small space like the iconic “garage” serving as a nucleus for later industrial complexes or multinational companies, examples of which are well known. In addition the people initiating such a development or starting such businesses not rarely are very individualistic characters. Universities belong to those rare institutions providing space and resources in which unique characters, for which the term “nerd” has become popular, are able to unfold creativity and realize odd projects in a specific way of combining life and work.

## Cultural differences

The knowledge transfer from universities (basic but, in part, also applied research, i.e., the “ivory tower”) to industry (i.e., in the meaning of earning money with applications) represents one of the repeatedly discussed borders. Within the scientific community a certain amount of disinterest exists with regard to the needs of industry or society. Confronted with these needs scientists like to claim the “freedom of research and teaching” for themselves. But it may also be the fear of control, to be under constraints or undue influence of industry, pressure on performance or more generally being exposed to critique eventually putting in question the relevance of someone’s activities.

Vice versa industry and/or society often show a considerable amount of ignorance with respect to the irritating thematic diversity and specific culture at universities, often referred to as “creative chaos” in a rather positive sense or, more negatively, inefficiency. If confronted with the nature of everyday scientific life (which may include lying on a sofa, pretending to think thoroughly or endless chatting without an agenda in contrast to sitting at an organised desk or attending a well-prepared and structured business meeting), people not familiar with this kind of working environment show a certain amount of helplessness, if not ignorance. In such situations statements are made that might contain phrases including those claiming that “these people are paid with public money” and that “their work should be of relevance to society or industry”, i.e., “return on investment” instead of “research in the ivory tower”. Such arguments are quickly and gratefully adopted by politicians as well, who prefer predictability over spontaneity and a manageable amount of topics over thematic variability and, finally and most important, control instead of free individualistic behaviour.

As a consequence universities or other research institutions may appear as a parallel world in contrast to industry although both aim at solving problems, however, with different aims and outcomes (Table 1):

**Table 1:** Differences in motivation to solve problems in industry and the scientific world.

industry	academia

economic success	satisfying personal curiosity
yields	third-party funding
shareholder value	impact points
market share	publications

**Figure 3:**
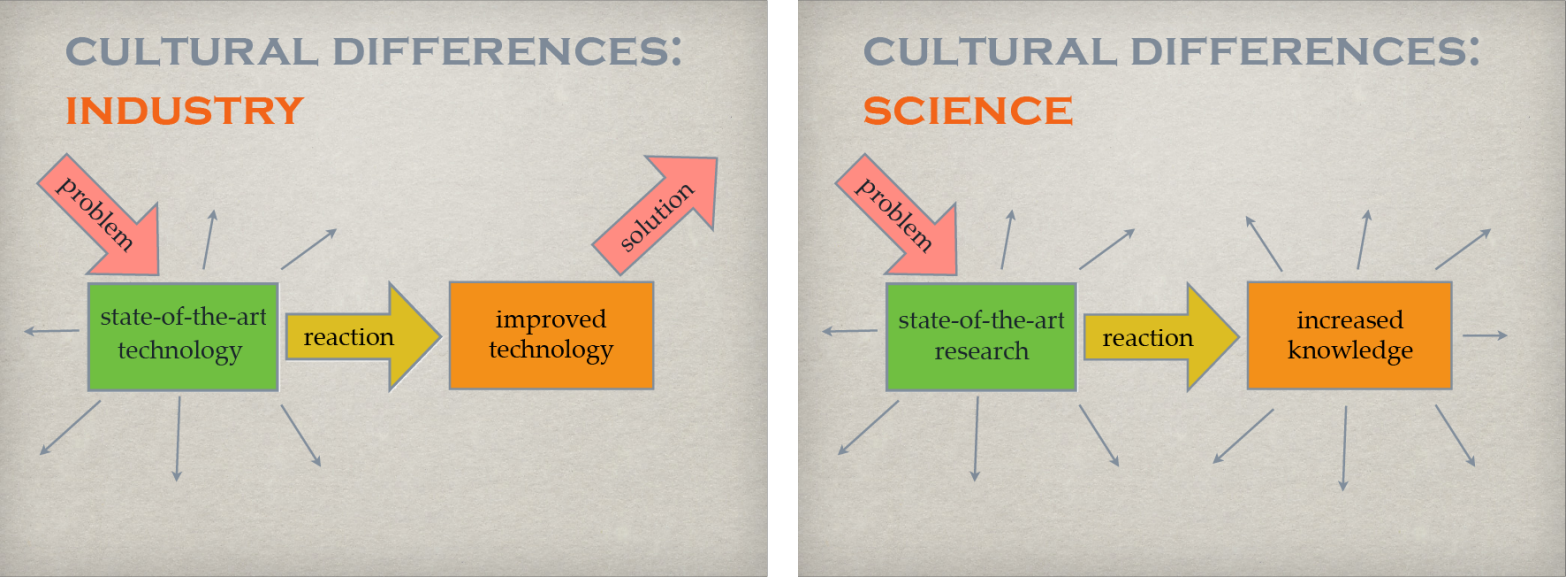
Some of the cultural differences between industry and science.

These differences in aims and interests are inherently connected to different methods, internal procedures, control mechanisms, or parameters for success (Figure 3). When faced with a problem, companies offering a given technology usually try to improve this technology. They aim to find a better solution securing a given success on the market, but not necessarily the best solution for the problem; although these paths to improve the technology are potentially open. The improvement preferentially happens at highest speed and lowest costs to maximise economic success, an approach that is a priori biased usually neglecting other, probably more successful solutions on a longer time scale.

The first reaction of scientists to a given problem is similar, in the way that an appropriate experimental setup will be chosen to solve this problem. In this respect the process may be comparatively biased as well. The outcome, however, is not a better product that may remain unchanged for a considerable amount of time, but a new problem, resulting in a new experiment, hypothesis, theory, or method and therefore increased knowledge in an iterative process. Both approaches have their pros and cons and one is not necessarily better than the other. Many companies, especially those active in information technology, try to establish similar environments for individuality to foster creative solution finding.

**Figure 4:**
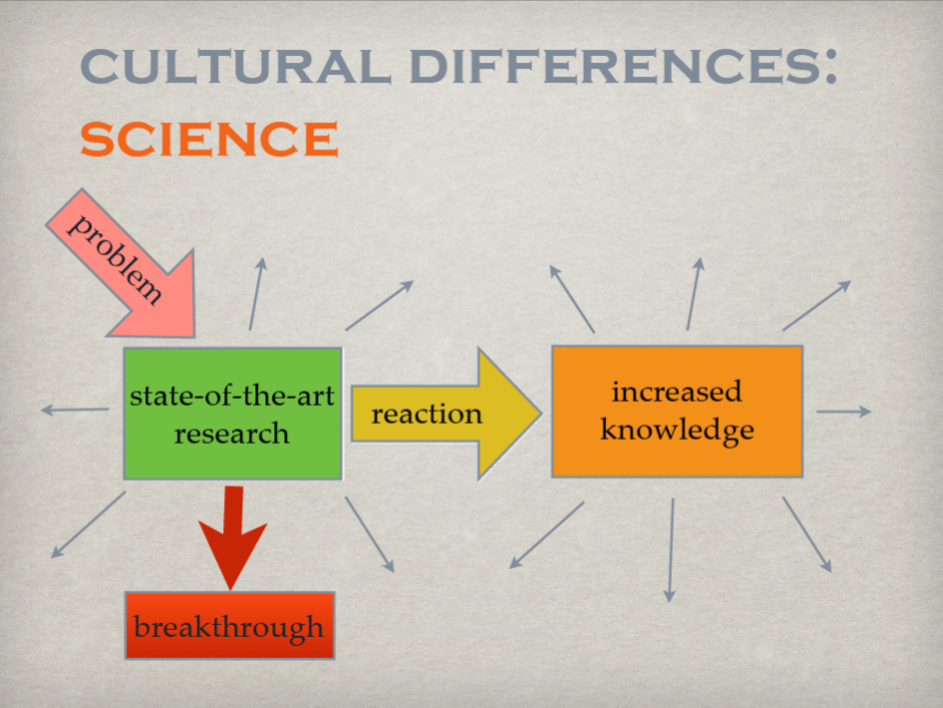
Achieving a breakthrough by following ideas off the mainstream.

Exploring different ways in parallel, although most of them may represent dead ends, is taken for granted in universities, and this is probably the most distinct difference between a company and a university lab. Nobody is surprised or will be blamed if an experimental setup fails or does not lead to the desired result (except the people who was running the experiment and needed the results). This is simply part of the job. Exploring all these possibilities, on the other hand, may result in unexpected and surprising results, eventually providing a technological, theoretical or methodological breakthrough. Not surprisingly, a considerable amount of such groundbreaking results derived from university research or institutions that are, at least partly, dedicated to basic research (Figure 4).

**Figure 5:**
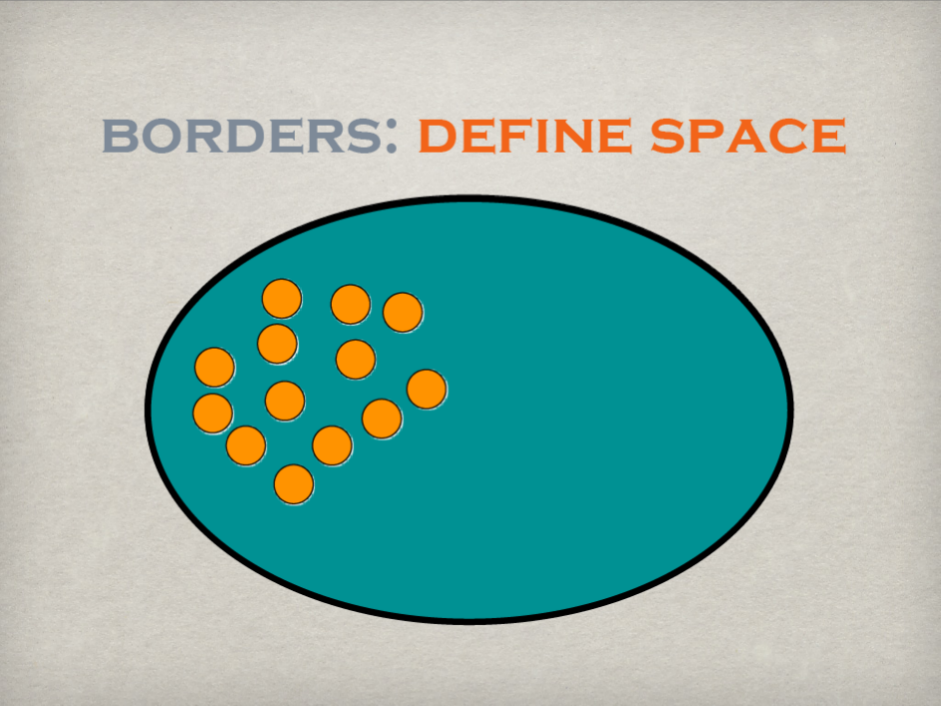
The available space for opportunities may be explored but does not necessarily need to be.

As mentioned above, borders define space and may represent limits that, at least at a given time, might be impossible to overcome. Claiming freedom of research inherently includes the duty to use this freedom, or to fill the available space (Figure 5). There might be temporary obstacles such as of technological nature as can be seen from computer industry in which computational power and storage capacity is increased on a regular basis by improved manufacturing processes. Others may be of ethical nature, such as the genetic engineering of microbes or the cloning of humans. A third obstacle may be intellectual property rights, hindering research and development. But all these obstacles are temporary. On the long run, everything that is possible will be realized, once an idea, a method, or a theory has become public and the technology is available. Not using the available space, however, allows for a niche existence without much attendance from the public or the scientific community. It allows for creating an “oasis of well-being” to avoid competition, to spend a nice time in a relaxed environment, a picture likely to be used by the public, politicians or business consultants invited to “optimise” structures and processes at universities.

## Selectivity

**Figure 6:**
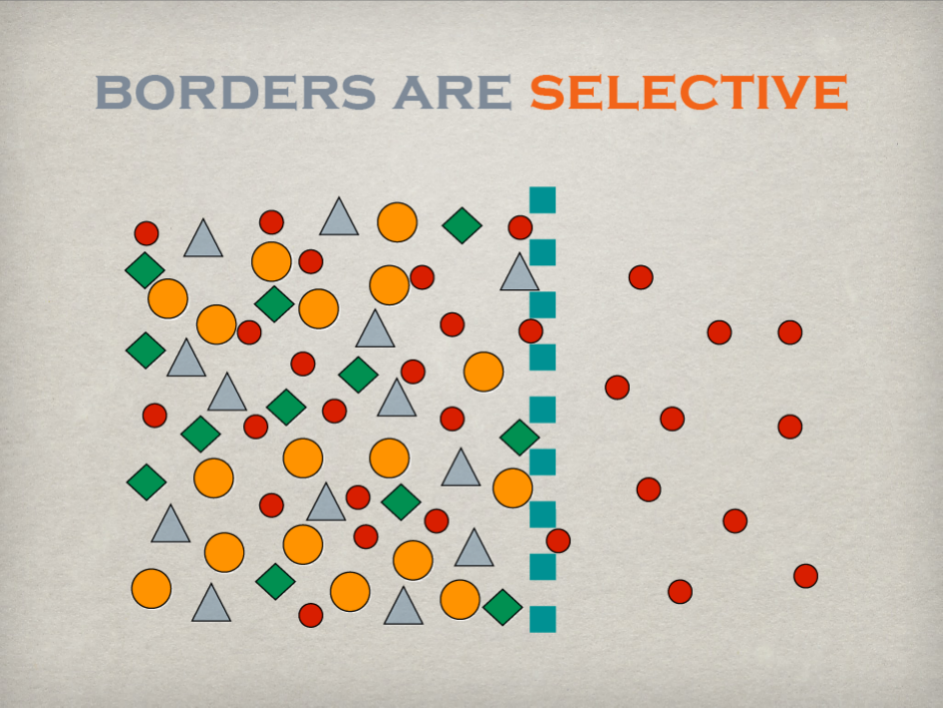
The selective permeability is another main function of borders.

Selectivity is another important feature of borders (Figure 6). There are hardly any impenetrable borders as such. Therefore, a certain amount of exchange will always take place. Selectivity may be related to the exchange of matter, e.g., semipermeable membranes of cells and organelles, to the exchange of information regulated by confidentiality agreements, or to the loss of energy minimised by insulating material fixed to a façade. In the relation between university and industry and/or society, selectivity may be represented by the amount of results published or communicated by the scientific community (“I decide to publish only what I want”). On the other hand, the demand from industry and/or society is highly selective as well. Only results and information regarded as “relevant” under the constraints of applied filters will be extracted from the huge pool of accumulated knowledge irrespective of what is available and offered (“give me only the information that I want”).

These constraints are, in particular, scientific relevance and a framework that is based, among other things, on evaluations, performance criteria, impact factors and h-index, and the amount of third-party funding. This framework arises from political specifications such as quality pacts or target agreements. Thus, opportunistic behaviour is rewarded (you will be funded, if…; the idea is nice, but the reviewer suggests that…; you could get more staff after adding…) and research is shaped according to external criteria; proposals are formulated to fit into program descriptions, self-censorship is becoming the second nature of scientists.

Most importantly, the resilience of the scientific system/community to respond to future challenges will be severely threatened by reducing diversity and mainstreaming research. This is probably one of the most important lessons to be learned from nature: With increasing diversity ecosystems seem to be more resilient against external influences and disturbance [1–2].

Along with the processes mentioned above other developments are emerging with consequences hard to predict, such as the equalisation of universities and Fachhochschulen (universities of applied sciences) in Germany. The repeatedly demanded and rewarded “relevance”, flanked by political measures, potentially allows industry to exploit universities. Even more, the expectations are that outstanding performance can be achieved without an appropriate investment into the institutions. Examples are the salaries of Ph.D. students and university staff, or the financial basis that governments are willing to provide for research and teaching at universities. The selective demand for results and information may result in a reduction of the diversity of research topics at universities on the one hand. On the other hand, the political framework suffocates creativity and will cause frustration by those not regarded as “relevant”. It may even lead to the sell-out of scientists on the border of self-abandonment. At the same time, exactly the same people claim that we need new ideas, innovative technologies and clever production tools to address the upcoming challenges for our societies, requiring out-of-the-box thinking, unconventional research approaches and sometimes weird stuff off the mainstream. One of these examples represents the early work of Wilhelm Barthlott.

## Systematic botany, epicuticular waxes and a paradigm shift in interface science

In the 1970th, a new era began for the young Ph.D. student Wilhelm Barthlott at the Institute for Botany of the University of Heidelberg. He received one of the first scanning electron microscopes in German botany and started to intensively study the fascinating world of micro- and nanostructures of leaves, flowers, seeds or pollen grains.

Starting point was a distinct interest in systematics, i.e., the science of recording and arranging organisms according to their relation to each other as well as their natural history in connection with a botanic garden, which was keeping extensive collections, being at his disposal. During his approach to conduct broad surveys among various groups of plants, he soon recognized that certain structures were not distributed randomly but characteristic for distinct genera, families or higher-order groups. One of the first structures studied in detail were seeds [3–8]. Apart from the sole description of structures based on the surveys functional aspects of plants were always considered as well [3,6,9–11].

Soon, and even more intensively, Wilhelm Barthlott concentrated on those minute structures on leaves, shoots, or flowers, called epicuticular waxes [12–28]. During these extensive surveys thousands of species have been characterised by scanning electron microscopy compiling, to our knowledge, the largest dataset on plant epicuticular surface features. Epicuticular waxes are made up of various soluble lipids and, at least most of them, originate from self-assembly, again a topic studied in various different types of crystals accompanied by recrystallization experiments and modern microscopy techniques [29–38].

**Figure 7:**
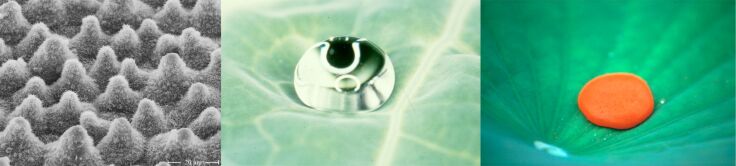
Fundamentals of self-cleaning in plants: a rough, hydrophobic surface (left) causes water to form spheres not adhering to the leaf (middle) removing particles while running off the leaf (right).

Examining plant surfaces, especially fine structures of micrometre size and smaller, through scanning electron microscopy needed careful preparation, including cleaning of the surfaces. After repeating these procedures again and again, Wilhelm Barthlott eventually realized that certain surfaces needed to be cleaned before examination while others did not. Surprisingly those surfaces that were already rather clean always turned out to be rough in certain dimensions and water-repellent, while those that were contaminated always were rather smooth or structured at a larger scale and readily wettable. He carried out simple experiments with *Tropaeolum majus* (Indian cress) by gluing small glass slides onto the surface of the leaves for a couple of weeks. A comparison of both surfaces, those of the leaves and those of the glass slides, revealed that the leaves were clean, while the glass slides were more or less densely covered by particles. Based on that observation, Wilhelm Barthlott formulated the hypothesis very early that “self-cleaning” might be one of the most important functions of rough water-repellent leaf surfaces [12] (Figure 7). This was elaborated in more detail later and the possibility of a technical application was already indicated [14].

Some years later, now appointed as professor for botany at the University of Bonn, Wilhelm Barthlott resumed this research. This was the time when I entered his group as student assistant. Based on numerous experiments revealing qualitative and quantitative data we were able to prove the astonishing self-cleaning properties of rough water-repellent surfaces by the end of the 1980s and realized that it was not published as a property of biological surfaces. Although this particular feature is easily observed and has nowadays become a standard experiment even in schools teaching bionics or biomimetics, it was virtually impossible to publish the results. Apart from an internal report of the University of Bonn [39] several attempts to publish the results failed. Finally, with the help of the former editor Andreas Sievers, the paper appeared in “Planta” five years after the first submission [40] followed by a survey about the characterisation and distribution of self-cleaning surfaces among plants [41]. Regardless of the scepticism from the scientific community, self-cleaning surfaces nowadays are well known. The transfer and technical application have received several awards and the trademark “Lotus-Effect” has become a kind of synonym for functional water-repellent or even only hydrophobic surfaces. Follow-up investigations have been published in all major journals and the original paper, until now, is cited on average every second or third day. A total number of several thousand articles dealing with micro- and nanostructured, water-repellent surfaces emphasises the significance of the original findings. Although the number of economically successful products featuring self-cleaning properties is rather limited, self-cleaning based on rough hydrophobic surfaces initiated a new field of research and represents a paradigm shift in interface science.

## So what happened and what can we derive?

**Figure 8:**
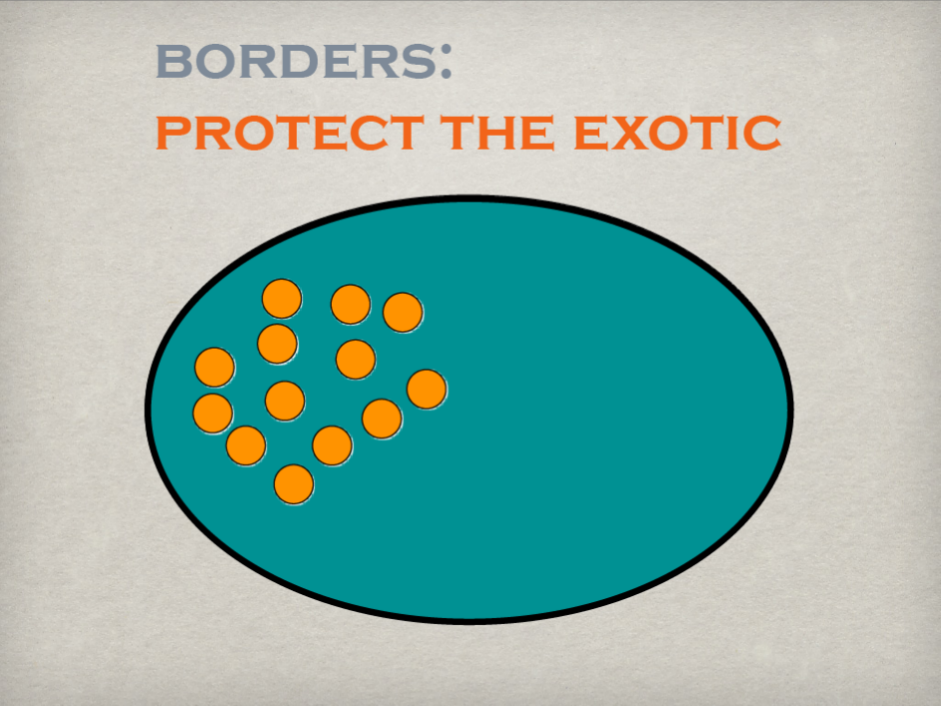
Borders separating a space from the surrounding may serve as a protective cover allowing for developments without external pressure or constraints.

Coming back to the picture of borders introduced above we may use self-cleaning surfaces as an example. First there is a field of research, systematic botany, often called “Orchideenfach” in German, i.e., an atypical academic discipline. Systematic botanists led a niche existence in their small academic world, were usually not very successful in raising third-party funding and published in journals with little or no impact at all and as such of limited public interest. This kind of existence, on the one hand, allows spending a whole scientific career, as mentioned above, in a kind of “oasis of well-being”, escaping competition, never proving any relevance for the society. On the other hand, research is conducted in a protected environment, without much pressure from outside, dealing with topics off the mainstream (Figure 8). This happens most probably in a much more open-minded community, in which unconventional solutions for problems are more likely found than in an environment with a much more biased research focus.

In this particular example, one result of the research was the answer to the question of the systematic affinities of sacred lotus (*Nelumbo nucifera*). For the longest time scientist considered water lilies (*Nymphaea*) to be the closest relatives of lotus. However, epicuticular waxes, small tubules mainly composed of the secondary alcohol nonacosan-10-ol, as well as a specific group of alkaloids, implied that poppies (Papaveraceae) were more likely the sistergroup [42], results which were independently substantiated by molecular data [43].

**Figure 9:**
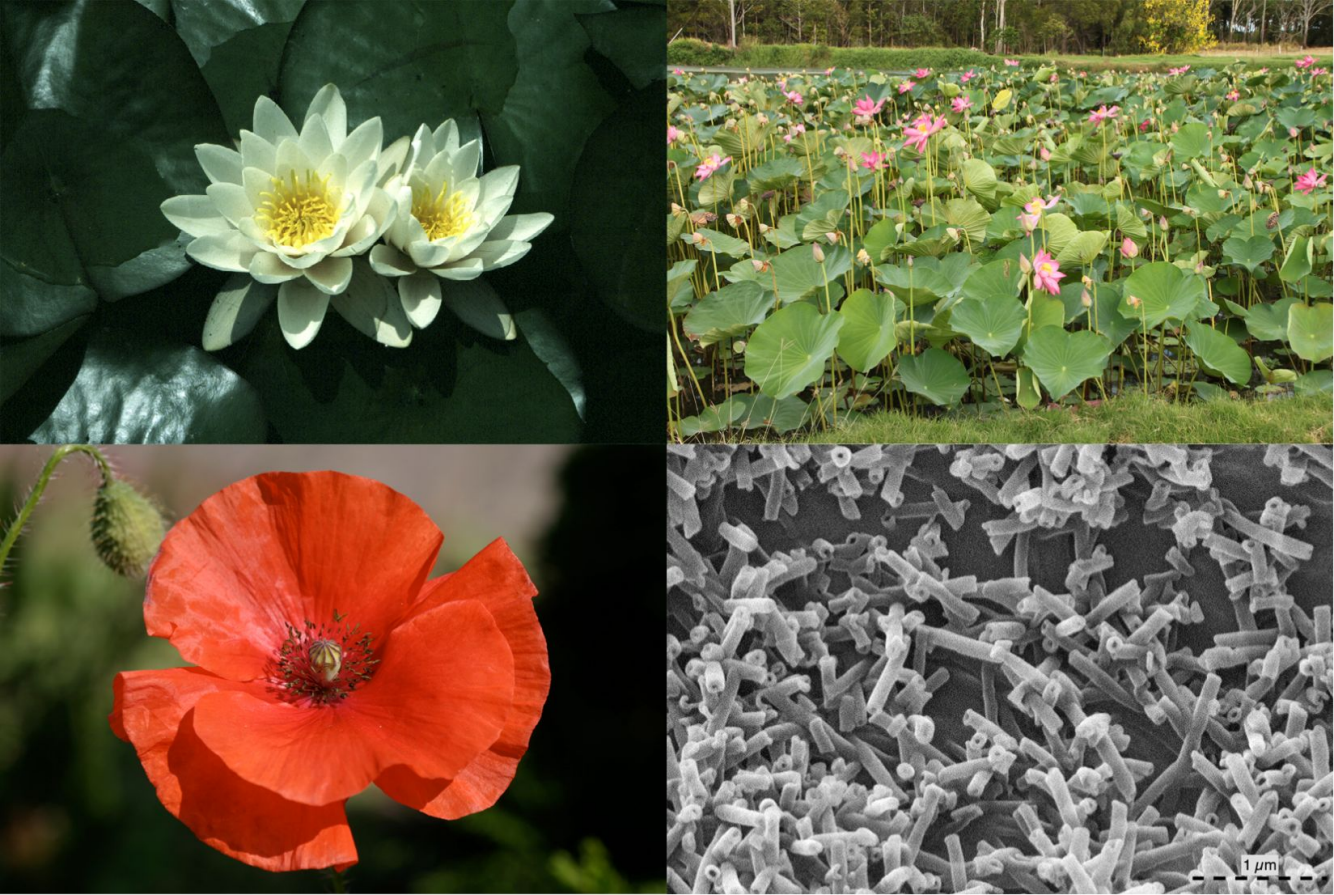
Is lotus related to water lilies (upper left) or poppies (lower left)? Epicuticular wax tubules (lower right) shared by ranunculids were one argument to place *Nelumbo* (upper right) close to the latter systematic group.

Nice, but who cares? Typical results that nobody is really interested in, virtually useless, and without hardly any practical use for industry. This is what business consultants use to call irrelevant and what the general public usually ignores. However, during these investigations a tremendous amount of data and knowledge about plant surfaces was accumulated that turned out to be essential for the later research on functional aspects of surfaces.

## Reluctance

After recognizing that self-cleaning in biological surfaces obviously was overlooked and realizing that a great potential for a transfer in technical surfaces existed, we contacted several companies introducing these fascinating properties to R&D departments. At that time we already cultivated quite a number of lotus plants for experiments and demonstrations allowing us to carry out simple experiments with leaves that were contaminated and cleaned by simply rinsing them with water.

Although everybody was electrified and instantaneously understood the principle and the potential, the outcome was frustrating. Again it was reluctance. The arguments were always the same: These are living organisms, much too complicated to understand and therefore it will be impossible to transfer the properties into a technical material. As a result, no cooperation could be established to move on and start a more practical project. Nobody was willing to invest in such a project and take the risk of failure.

**Figure 10:**
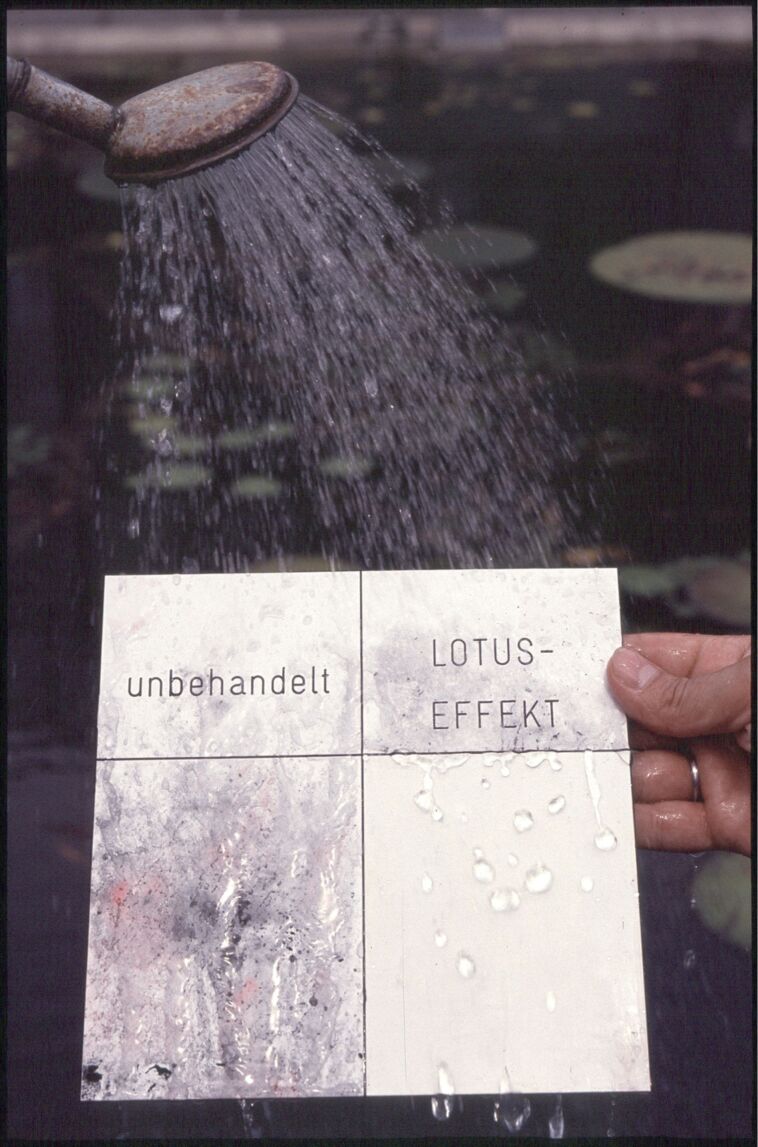
First demonstrator exhibiting the principle of self-cleaning derived from lotus leaves.

The picture changed fundamentally after we decided to produce some simple technical surfaces that provided the basic requirements for self-cleaning, namely a hydrophobic material and a certain roughness. Several attempts to reproduce lotus surfaces by embossing and other more sophisticated methods failed because of inappropriate technical skills and machinery. The first successful approach was rather simple: a commercially available polymer plate, which we covered with epoxy-resin glue fixing a layer of subsequently applied microscopic particles of PTFE. Although rather unstable with respect to mechanical influences, the plates exhibited the same properties as the lotus leaves. Figure 10 shows one of these early attempts. The plate has two sides, one smooth and one covered with PTFE particles. After contaminating both sides with toner from a photocopier and the red staining powder Sudan III, the plate was briefly rinsed with water. While the smooth side retained a considerable amount of particles, the structured one was completely cleaned. When we demonstrated this to several companies the reaction was completely different and the concept found acceptance.

Coming back to the picture that was introduced above, the following happened: Both the information demand from the industrial side as well as well as the information offer from the university side were filtered according to previous experience and knowledge . By realising these simple demonstrators the quality of information provided by the university changed and this in turn induced a different reception.

## The filter changed

The pore size of the filter changed, the border became permeable for a different type and quality of information because it was not “complicated biology” anymore but the property of self-cleaning was now demonstrated with a material that people in the industry were familiar with.

**Figure 11:**
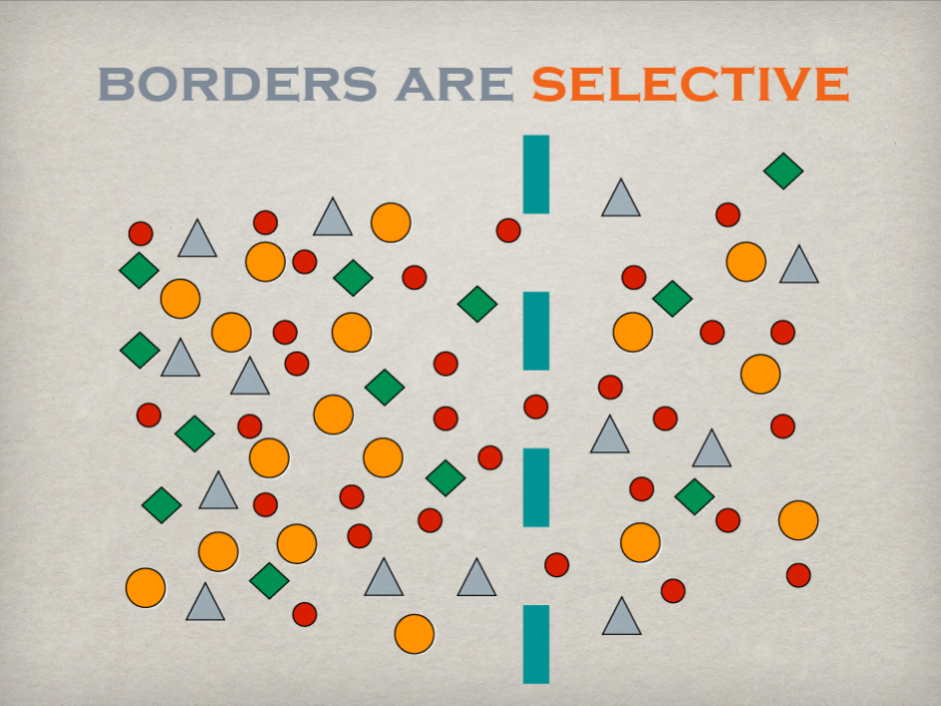
Depending on new developments, or changes in perception the selection criteria and, as a result, the permeability of the border may change .

And now we received the attention we were looking for and much more because the whole effect could be easily demonstrated, transported strong pictures and everybody who saw a demonstration was sure to have understood the principle. After that, the interest of companies from all kinds of fields was tremendous and a new kind of research was initiated. Although functional surfaces have been a topic of great interest and importance before, the whole field received a boost that still holds on today.

And another result became apparent, although quite a while later: the acceptance of bioinspired technology. Although self-cleaning surfaces were not the first technically applied inspirations derived from biological models – well-known examples are evolutionary algorithms [44–45], “winglets” at the tips of airplane wings [46], “riblets” derived from shark skins [47], or form optimization of components based on tree growth [48] –, biomimetic approaches are still regarded as exceptional and not suitable to serve as examples for a general approach. Although at least some of them, such as Claus Mattheck´s computer-aided optimisation and other methods, have been widely applied in engineering.

Although the number of commercially available products exhibiting self-cleaning properties based on rough hydrophobic surfaces similar to those of lotus leaves is rather limited, the field was opened and there was a positive reception from the general public, politics and, most important, industry. Paving the way and initiating all these new developments is probably the biggest achievement of this irrelevant little research project, which started about 40 years ago. The niche has become mainstream, the orchid has become relevant.

## Conclusion

Not everything that appears to be exotic and strange is irrelevant. Diversity, in all its various aspects should be maintained, even at high cost. Environments, be it biological, political, or economical change. This is their nature and we need to be prepared. We do not know the solutions for future challenges, we even do not know the challenges, but they will appear sooner or later and, who knows, may be some eccentric, spleeny young student is already puzzling about the solution.

This is a plea for the largest diversity possible under given circumstances in research and teaching. A reclusive existence in a niche at a university institute, a museum or even a lab off the beaten track in a company might imply snugness or laziness of the inhabitants. But it also means a little less conformity, a little bit more freedom, disorder, creativity and frankness for unconventional approaches. We need both worlds, both kinds of people, approaches and mentalities.

Wilhelm Barthlott represents one of these open-minded, unconventionally thinking people, off the beaten track who influenced the nature of many borders and paved new paths in the field of functional surfaces and other research areas. As mentioned above this was only one of his research topics among many that I did not mention. His contributions to the systematics of Cactaceae, the first comparative studies of rocky outcrops called “inselbergs” on a global scale, and finally the mapping of the global biodiversity including his long-lasting and continuing plea for the conservation of the whole of biological diversity will remain incentive and duty for future scientists.
